# SARS-CoV2 infection and primary school closure

**DOI:** 10.2807/1560-7917.ES.2020.25.15.2000617

**Published:** 2020-04-16

**Authors:** Philippe Vanhems

**Affiliations:** 1 Hospices Civils de Lyon, Lyon 1 University and International Center for Research in Infectiology (CIRI), Lyon, France

**Keywords:** SARS-CoV-2 School closure Transmission Children Control Social contacts

**To the editor: **The recent review of Brooks et al. on the impact of unplanned school closure on children’s social contact [1] for controlling outbreaks brings interesting information that could apply to coronavirus disease (COVID-19). In addition, a recent case in France of a 9-year-old child infected by SARS-CoV-2 [2] raises the issue of risk assessment for other children at a same school and/or in a same classroom.

Identification of contacts between classmates is of high importance for appropriate screening and implementation of preventive measures at a primary school level but also at a family level. It has been reported that the patterns of contacts strongly differ according to age and school grade. For example, based on radio-frequency identification devices (RFID) technology, it was reported that young French children (age 6 years) in a primary school [3] had a median of 500 contacts per school day and a median of 300 minutes of cumulated contact per day. Older children (age 10–11 years) had a median of 300 contacts per day and a median of 250 minutes of cumulated contact per day. An aggregate analysis emphasised that young children interacted with many schoolmates of the same or similar age (age 7–8 years) while older children restricted their contacts mostly to their own age stratum, like in England [4].

The practical application of such an observation would therefore be to help public health authorities identify the children at higher risk of exposure. The decision to close a school totally or partially according to the age of an index case should be discussed. However, in an emergency context such as the COVID-19 pandemic where scientific knowledge regarding the virus is still lacking, total closure of a school was reasonable and reassuring for parents.

Management of such an event raises two issues in public health decisions. On the one hand, an understandable precautious public health decision for total school closure, and on the other hand, a detailed risk assessment with a potentially different decision. Although SARS-CoV-2 is not influenza or a respiratory syncytial virus, previous studies have identified the major impact of different social contacts of children by age which could have an impact on the spread of respiratory viral infections in schools [5]. Attack rates would differ according to grade or age, which determine the different contact patterns between children and would make it possible to adapt infection control measures [6]. However, a more discriminant risk estimation by age at onset of a public health emergency would appear not to be useful but might be helpful regarding strategies of re-opening schools with sequential access to courses. Nevertheless, at least retrospectively, detailed analysis of inter-individual contact remains a key determinant with viral characteristics in order to understand the dynamic of viral transmission in close environments such as primary schools.

## References

[1] Brooks SK Smith LE Webster RK Weston D Woodland L Hall I The impact of unplanned school closure on children’s social contact: rapid evidence review. Euro Surveill. 2020;25(13):2000188. 10.2807/1560-7917.ES.2020.25.13.2000188 32265006 PMC7140596

[2] Sante Publique France. Infection au nouveau Coronavirus (SARS-CoV-2), COVID-19, France et Monde. [Infections with novel coronavirus (SARS-CoV-2), COVID-19, France and worldwide]. Paris: Sante Publique France. [Accessed 14 Apr 2020]. French. Available from: https://www.santepubliquefrance.fr/maladies-et-traumatismes/maladies-et-infections-respiratoires/infection-a-coronavirus/articles/infection-au-nouveau-coronavirus-sars-cov-2-covid-19-france-et-monde

[3] Stehlé J Voirin N Barrat A Cattuto C Isella L Pinton JF High-resolution measurements of face-to-face contact patterns in a primary school. PLoS One. 2011;6(8):e23176. 10.1371/journal.pone.0023176 21858018 PMC3156713

[4] Conlan AJ Eames KT Gage JA von Kirchbach JC Ross JV Saenz RA Measuring social networks in British primary schools through scientific engagement. Proc Biol Sci. 2011;278(1711):1467-75. 10.1098/rspb.2010 21047859 PMC3081745

[5] Potter GE Handcock MS Longini IM Jr Halloran ME Estimating within school contact networks to understand influenza transmission. Ann Appl Stat. 2012;6(1):1-26. 10.1214/11-AOAS505 22639701 PMC3359895

[6] Gemmetto V Barrat A Cattuto C Mitigation of infectious disease at school: targeted class closure vs school closure. BMC Infect Dis. 2014;14(1):695. 10.1186/s12879-014-0695-9 25595123 PMC4297433

